# Virulence Characteristic and MLST-*agr* Genetic Background of High-Level Mupirocin-Resistant, MRSA Isolates from Shanghai and Wenzhou, China

**DOI:** 10.1371/journal.pone.0037005

**Published:** 2012-05-18

**Authors:** Qingzhong Liu, Lizhong Han, Bin Li, Jingyong Sun, Yuxing Ni

**Affiliations:** 1 Department of Clinical Laboratory, Shanghai First People’s Hospital, School of Medicine, Shanghai Jiaotong University, Shanghai, China; 2 Department of Clinical Microbiology, Ruijin Hospital, School of Medicine, Shanghai, China; University of Iowa, United States of America

## Abstract

The emergence and prevalence of high**-**level mupirocin**-**resistant, methicillin**-**resistant *Staphylococcus aureus* (MuH MRSA) is challenging the eradication of MRSA nasal carriage and the treatment of skin and soft tissue infections. To understand the potentially pathogenetic capacity and the genetic basis of MuH MRSA, it is important to have a detailed knowledge of the molecular traits of this organism. Fifty three MuH MRSA isolates were gathered from Shanghai (28 isolates) and Wenzhou (25 isolates) in China. These isolates, consisting of 27 different PFGE-SCC*mec*-*spa* patterns, were examined by PCR for 35 virulence genes and further typed using *agr* (accessory gene regulator) typing and MLST (multilocus sequence typing). All 53 strains were positive for the genes *hlg*/*hlg* variant and *icaD*, and negative for *seb*, *sed*, *see*, *seh*, *eta*, *etb*, *hld*, *cap-5*, and *ACME*-*arcA*. Compared with Wenzhou isolates, Shanghai isolates were more likely to carry *seg* (*P* = 0.002) and several other genes which were not found in Wenzhou strains such as *sec*, *sei*, *tst* (*P*<0.001 each), and *pvl* (*P* = 0.012), and less likely to contain *sea* (*P*<0.001), *cna* (*P* = 0.031), and *efb* (*P* = 0.045). MLST and *agr* typing showed that ST239-*agr1*, ST5-*agr1*, and ST239-*agr2* were the common lineages in MuH MRSA isolates from these two different regions. Our results indicated that MuH MRSA strains from two different geographic regions of China have differences in distribution of some virulence genes, while their major MLST-*agr* genetic backgrounds were accordant.

## Introduction

Methicillin-resistant *Staphylococcus aureus* (MRSA) is still a leading pathogen of nosocomial infections in China. Because of its ability to produce virulence factors causing a variety of infections and its serious multidrug resistance, MRSA is one of the most frightening bacteria.

The pathogenesis of *S. aureus* infections is involved in the expression of a wide range of virulence factors associated with attachment, persistence, evading/destroying host defenses, tissue invasion/penetration and toxin**-**mediated disease [1]. However, a majority of the serious *S. aureus* infections are caused by the combined actions of several virulence factors. Otherwise, clinical isolates linked to *S. aureus* infection may be naturally deficient in a scale of putative pathogenic determinants. Therefore, strains causing *S. aureus* disease have variable combinations of virulence genes [2].

Mupirocin is a very effective topical antibiotic used to treat staphylococcal skin and soft tissue infections and eliminate MRSA from colonized nasal passages. However, the resistance to mupirocin has occurred, and its spreading is worrisome, with *mupA* **-**mediated high**-**level resistance [3]. The *mupA* gene is usually located on a large conjugative plasmid capable of mediating the co-transfer of other resistance genes. If high-level mupirocin resistance could not be controled, a highly effective means of decolonization of MRSA may be lost. Thus the high-level mupirocin resistance MRSA (MuH MRSA) may spread widely, and causes a range of infections. Therefore it is necessary to know the traits of virulence of clinical MuH MRSA strains more comprehensively.

The purpose of this study was to investigate the prevalence of putative virulence genes in a clinical population of MuH MRSA isolates with known types of PFGE-SCC*mec*-*spa* from two different geographic areas in China, and explore the possible difference in virulence determinants combination between the strains from the both origins. Finally we further determined the genetic characteristics of these isolates by *agr* (accessory gene regulator) typing and multilocus sequence typing (MLST).

## Materials and Methods

### MuH MRSA Isolates

Fifty three MuH MRSA isolates were selected from 5 university hospitals collection in Shanghai (n = 28, 4 hospitals) and Wenzhou (n = 25, 1 hospital), China. This collection comprised 803 MRSA, which were isolated from various clinical specimens of individual inpatients from August 2005 to May 2008. Most of the MuH isolates were gathered from respiratory samples (86.8%). Intensive care units, surgical wards, burn wards and neurology wards were the major hospital units affected by MuH MRSA. All isolates had been previously described by PFGE, SCC*mec* and *spa* typing and these results are shown in Table 1 [4]. Because of being focused on bacteria, this study was exempted from review by the Ethics Committee of Shanghai First People’s Hospital.

**Table 1:** The PFGE-SCC*mec-spa* patterns of 53 MuH MRSA isolates [4].

PFGE-SCC*mec-spa*type	No. of isolates (n)	Originationof isolates	PFGE-SCC*mec-spa*type	No. ofisolates (n)	Originationof isolates
A1-IIIA-t030	12	Wenzhou	I-III-t037	3	Shanghai
A2-IIIA-t030	4	Wenzhou	J-I-t189	1	Wenzhou
A3-IIIA-t030	1	Wenzhou	K-III-nontypeable	1	Shanghai
A4-IIIA-t030	1	Wenzhou	L1-II-t002	1	Shanghai
A5-IIIA-t037	2	Shanghai	L2-III-t002	1	Shanghai
B-IIIA-t030	4	Wenzhou	M1-IIIA-t002	2	Shanghai
C-IIIB-t037	1	Shanghai	M2-IIIA-t002	1	Shanghai
D1-IIIA-t037	1	Shanghai	N-I-t318	5	Shanghai
D2-IIIA-t037	1	Shanghai	N-IA-t318	1	Shanghai
E-IIIA-t127	1	Shanghai	O-III-t377	1	Shanghai
F1-II-t002	1	Shanghai	P1-III-t037	2	Shanghai
F2-II-t002	1	Shanghai	P2-III-t037	1	Shanghai
G-IIIA-t030	1	Wenzhou	Q-IIIA-t985	1	Shanghai
H-IIIA-t2505	1	Wenzhou			

### Detection of Virulence Factor Genes and *agr* Alleles

Genomic DNA from MuH MRSA was extracted by the bacterial genomic DNA kit (Wuxi Institution of Clone and Genetic Technology, China) and then used for PCR amplification of 35 virulence genes (involved in toxin mediated disease, attachment, evading/destroying host defenses, tissue invasion/penetration, persistence and others) and *agr* alleles (allele *1* to *4*) by the primers derived from the published sequences [2], [5]-[14]. These genes were all listed in Table 2.

**Table 2:** The genes detected by PCR in this investigation.

Virulence gene	Reference	Virulence gene	Reference
Involved in toxin mediated disease and/or sepsis		Capsular polysaccharide 5, 8 (*cap5*, *cap8*)	9
Staphylococcal enterotoxin A, B, C, D, E, G, H, I, J(*sea*, *seb*, *sec*, *sed*, *see*, *seg*, *seh*, *sei*, *sej*)	5,6,7	Major-histocompatibility-complex class II-analogue protein (*map*)	2
Exfoliative toxin A, B (*eta*, *etb*)	5	IgG**-**binding protein SBI (*sbi*)	10
α, β, δ-hemolysin (*hla*, *hlb*, *hld*)	5	Involved in tissue invasion/penetration	
Toxic shock syndrome toxin-1 (*tst*)	6	V8 serine protease (*ssp*)	10
Involved in attachment		Staphylokinase (*sak*)	11
Fibronectin-binding protein A, B (*fnbA*, *fnbB*)	2,8	Involved in persistence	
Clumping factor A,B (*clfA*, *clfB*)	2	Intercellular adhesion A, D (*icaA*, *icaD*)	12
Collagen adhesin (*cna*)	2	Others	
Bone sialoprotein**-**binding protein (*bbp*)	2	Staphylococcal accessory regulator A (*sarA*)	10
Elastin-binding protein (*ebpS*)	2	Extracellular fibrinogen**-**binding protein (*efb*)	10
Involved in evading/destroying host defenses		* arcA* region of arginine catabolic mobile element (ACME-*arcA*)	13
Panton-Valentine leukocidin (*pvl*)	5	*agr* alleles	
γ-hemolysin and variant (*hlg*, *hlgv*)	5	allele *1* to *4*	14

### DNA Sequencing

One randomly chosen amplicon for the each gene tested was sequenced on an ABI 3730 sequencer (Applied Biosystems) by Shanghai Invitrogen Biotech to confirm that primers amplified the expected genes.

### MLST

MLST was performed on 27 MuH MRSA isolates representative of each PFGE-SCC*mec*-*spa* type as described previously [15]. Sequence type (ST) of each strain was determined by sequencing internal fragments of 7 housekeeping genes (*arcC*, *aroE*, *glpF*, *gmk*, *pta*, *tp*i and *yqiL*) according to the MLST database (http://www.mlst.net).

### Statistical Analyses

Pearson’s chi-square test or Fisher’s exact test if necessary was used to compare distribution of the virulence determinants in clinical MuH MRSA strains investigated (SPSS version11.5). All statistical tests were two tailed, with *P*<0.05 considered statistically significant.

## Results

### Comparison of Virulence Genes in Shanghai and Wenzhou Isolates

Compared to Wenzhou isolates, Shanghai strains were significantly less likely to contain *sea* (32.1% versus 80.0%, *P*<0.001), *cna* (67.9% versus 92.0%, *P* = 0.031) and *efb* (64.3% versus 88.0%, *P* = 0.045), and more likely to host *seg* (57.1% versus 16.0%, *P* = 0.002). For Shanghai strains, 42.9%, 53.6%, 50.0%, 7.1% and 28.6% carried *sec*, *sei*, *tst*, *sej* and *pvl*, respectively, whereas no isolate from Wenzhou possessed them, and those differences except that of *sej* were significant. No isolate from either origin was positive for *seb*, *sed*, *see*, *seh*, *eta*, *etb*, *hld*, *cap-5* and *ACME*-*arcA* (Table 3). The distribution of the remaining virulence genes in 53 MuH MRSA isolates were summarized in Table 3.

**Table 3:** Distribution of 35 virulence genes among the isolates of MuH MRSA from Shanghai and Wenzhou, China.

Gene	No. of isolates positive for the gene [% of total (n = 53)]	No.of isolates positive for the gene in two regions (%)			χ^2^ (*P* value)
		Shanghai (n = 28)	Wenzhou (n = 25)		
Involved in toxin mediated disease and/or sepsis					
*sea*	29 (54.7)	9 (32.1)	20 (80.0)		12.208 (<0.001)
*seb*	0	0	0		NA
*sec*	12 (22.6)	12 (42.9)	0		13.850 (<0.001)
*sed*	0	0	0		NA
*see*	0	0	0		NA
*seg*	20(37.7)	16 (57.1)	4 (16.0)		9.515 (0.002)
*seh*	0	0	0		NA
*sei*	15 (28.3)	15 (53.6)	0		18.680 (<0.001)
*sej*	2 (3.8)	2 (7.1)	0		(0.492)*
*tst*	14 (26.4)	14 (50.0)	0		16.987 (<0.001)
*eta*	0	0	0		NA
*etb*	0	0	0		NA
*hla*	52 (98.1)	27 (96.4)	25 (100)		(1.000)*
*hlb*	43 (81.1)	21 (75.0)	22 (88.0)		0.733 (0.392)
*hld*	0	0	0		NA
Involved in attachment					
*fnbA*	48 (90.6)	26 (92.9)	22 (88.0)		0.018 (0.894)
*fnbB*	8 (15.1)	7 (25.0)	1 (4.0)		3.045 (0.081)
*clfA*	41 (77.4)	21 (75.0)	20 (80.0)		0.189 (0.664)
*clfB*	52 (98.1)	27 (96.4)	25 (100)		(1.000)*
*cna*	42 (79.2)	19 (67.9)	23 (92.0)		4.681 (0.031)
*bbp*	7 (13.2)	6 (21.4)	1 (4.0)		2.145 (0.143)
*ebpS*	50 (94.3)	27 (96.4)	23 (92.0)		0.010 (0.919)
Involved in evading/destroying host defenses					
*pvl*	8 (15.1)	8 (28.6)	0		6.331 (0.012)
*hlg*	46 (86.8)	22 (78.6)	24 (96.0)		2.145 (0.143)
*hlgv*	7 (13.2)	6 (21.4)	1 (4.0)		2.145 (0.143)
*cap-5*	0	0	0		NA
*cap-8*	48 (90.6)	23 (82.1)	25 (100)		3.061 (0.080)
*map*	44 (83.0)	23 (82.1)	21 (84.0)		0.000 (1.000)
*sbi*	51 (96.2)	28 (100)	23 (92.0)		(0.218)*
Involved in tissue invasion/penetration					
*ssp*	46 (86.8)	23 (82.1)	23 (92.0)		0.425 (0.515)
*sak*	45 (84.9)	21 (75.0)	24 (96.0)		3.054 (0.081)
Involved in persistence					
*icaA*	43 (81.1)	21 (75.0)	22 (88.0)	0.733 (0.392)	
*icaD*	53 (100)	28 (100)	25 (100)	NA	
Others					
*sarA*	46 (86.8)	24 (85.7)	22 (88.0)	0.000 (1.000)	
*efb*	40 (75.5)	18 (64.3)	22 (88.0)	4.012 (0.045)	
*ACME*-*arcA*	0	0	0	NA	

*P*<0.05 were considered statistically significant.

NA, not available.

* Fisher’s exact test.

### Virulence Genes Content and Combination in Shanghai and Wenzhou Isolates

Of 28 Shanghai strains, 13 (46.4%) possessed ≥5 virulence genes involved in toxin mediated disease (high toxin gene content), however, no Wenzhou strain carried ≥5 this type of genes. The most prevalent combination of this type of genes was *hla*+*hlb*+*seg*+*sei*+*tst*+*sec* (25.0% of strains, 7/28) in Shanghai strains. And the second frequent combination was *sea*+*hla*+*hlb* (17.9% of strains, 5/28), which was also the most main combination in Wenzhou strains (68.0% of strains, 17/25) (Table 4). As for the adhesion determinants, 17 (60.7%) Shanghai isolates and 20 (80.0%) Wenzhou isolates possessed ≥5 genes. The combinations of *fnbA*+*clfA*+*clfB*+*ebpS*+*bbp*+*cna* and *fnbA*+*clfA*+*clfB*+*ebpS*+*cna* were overrepresented in Shanghai (21.4%, 6/28) and Wenzhou strains (76.0%, 19/25), respectively (Table 4). As indicated in Table 4, there were 8 (28.6%, 8/28) Shanghai iaolates harboring the pattern of *hlg*+*sbi*+*cap-8*+*map*+*pvl*, and 20 (80.0%, 20/25) Wenzhou isolates containing the combination of *hlg/hlgv*+*sbi*+*cap-8*+*map*. However, no Wenzhou strain hosted ≥5 this kind of determinants. The other combination of virulence genes were shown in Table 4.

**Table 4:** Virulence gene profile and genetic characteristics of 53 MuH MRSA isolates from Shanghai and Wenzhzou, China.

Strain	Virulence gene profile						*agr*type	PFGE-SCC*mec*-*spa* ^#^ [4]	MLSTtype*
	Involved in toxin mediated disease and/or sepsisdisease and/or sepsis	Involved in attachment	Involved in evading/destroying hostdefenses	Involved in tissue invasion/penetration invasion/penetration invasion/penetration invasion/penetration	Involved in persistence	Others			
Shanghai strain									
1, 3, 4, 5, 6	*hla, hlb, seg*, *sei*, *tst, sec*	*fnbA*, *clfA*, *clf*B, *ebpS*, *bbp*, *cna*	*hlg, sbi, cap-8*, *map*, *pvl*	*ssp*, *sak*	*icaD, icaA*	*sarA*	3	N-I-t318	ST284
2	*hla, hlb, seg*, *sei*, *tst, sec*	*fnbA*, *clfA*, *clf*B, *ebpS*, *bbp*, *cna*	*hlg, sbi, cap-8*, *map*, *pvl*	*ssp*, *sak*	*icaD, icaA*	*sarA*	3	N-IA-t318	ST284
7	*hla*, *hlb, seg, sei*, *tst*, *sec*, *sej*	*fnbA*, *clfA*, *clfB*, *ebpS*	*hlg, sbi, cap-8*, *map*	*ssp, sak*	*icaD, icaA*	*sarA, efb*	1	L1-II-t002	ST5
15	*hla, seg, sei, sej*	*fnbA, clfA, clfB, ebpS*	*hlg, sbi, map*	*ssp*	*icaD*	*sarA, efb*	2	L2-III-t002	ST5
8	*hla, hlb, sea*	*fnbA, clfA, clfB, ebpS, cna, fnbB*	*hlg, sbi, cap-8, map*	*ssp, sak*	*icaD, icaA*	*sarA, efb*	1	P2-III-t037	ST239
9, 11	*hla, hlb, sea*	*fnbA, clfA, clfB, ebpS, cna, fnbB*	*hlg, sbi, cap-8, map*	*ssp, sak*	*icaD, icaA*	*sarA, efb*	1	P1-III-t037	ST239
10	*hla*	*fnbA, clfB, fnbB*	*hlgv, sbi, cap-8, map*	*ssp, sak*	*icaD, icaA*	*sarA, efb*	1	O-III-t377	ST630
12	*hlb*	*fnbA, clfB, ebpS, cna*	*hlgv, sbi*	*ssp, sak*	*icaD*		2	I-III-t037	ST239
13	*hla*	*fnbA, ebpS, cna*	*hlgv, sbi*		*icaD*		2	I-III-t037	
14	*hla*	*fnbA, clfB, ebpS, cna*	*hlgv, sbi*		*icaD*		2	I-III-t037	
16	*hla, tst*	*clfB, ebpS,*	*hlgv*, *sbi, cap-8*		*icaD*		2	K-III-nt	ST239
17	*hla, hlb, seg, sei*	*fnbA, clfA, clfB, ebpS, fnbB*	*hlg, sbi, cap-8, map*	*ssp*	*icaD, icaA*	*sarA, efb*	1	D1-IIIA-t037	ST239
26	*hla, sea*	*fnbA, clfA, clfB, ebpS, cna*	*hlg, sbi, cap-8, map*	*ssp, sak*	*icaD, icaA*	*sarA, efb*	1	D2-IIIA-t037	ST239
18	*hla, hlb, seg, sei, tst, sec*	*fnbA, clfA, clfB, ebpS*	*hlg, sbi, cap-8, map*	*ssp*	*icaD, icaA*	*sarA, efb*	1	M2-IIIA-t002	ST5
23	*hla, hlb, seg, sei, tst, sec, sea*	*clfA, clfB, ebpS, cna*	*hlg, sbi, cap-8, map, pvl*	*ssp, sak*	*icaD, icaA*	*sarA, efb*	1	M1-IIIA-t002	ST5
24	*hla, hlb, seg, sei, tst, sec, sea*	*fnbA, clfA, clfB, ebpS, cna*	*hlg, sbi, cap-8, map*	*ssp, sak*	*icaD, icaA*	*sarA, efb*	1	M1-IIIA-t002	
19	*hla, hlb, sea*	*fnbA, clfA, clfB, ebpS, cna*	*hlg, sbi, cap-8, map*	*ssp, sak*	*icaD, icaA*	*sarA, efb*	3	E-IIIA-t127	ST1
20	*hla, hlb, seg, sei, tst*	*clfA, clfB, bbp, ebpS, cna*	*hlg, sbi, cap-8*	*ssp, sak*	*icaD, icaA*	*sarA, efb*	1	F2-II-t002	ST5
28	*hla, hlb*	*fnbA, clfA, clfB, ebpS*	*hlg, sbi, map*	*ssp, sak*	*icaD, icaA*	*sarA, efb*	1	F1-II-t002	ST5
21	*hla, hlb, seg, tst, sec*	*fnbA, clfA, clfB, ebpS, fnbB*	*hlg, sbi, cap-8, map, pvl*	*ssp, sak*	*icaD, icaA*	*sarA, efb*	1	C-IIIB-t037	ST239
22	*hla, hlb, seg, sei, tst, sec, sea sea,*	*fnbA, clfA, clfB, ebpS, cna, fnbB*	*hlg, sbi, map*	*ssp, sak*	*icaD, icaA*	*sarA, efb*	1	A5-IIIA-t037	ST239
27	*hla, hlb, sea*	*fnbA, clfB, ebpS, cna*	*hlg, sbi, cap-8, map*	*ssp, sak*	*icaD*	*sarA, efb*	1	A5-IIIA-t037	
25	*hla, seg, sei*	*fnbA, clfB, ebpS, cna*	*hlgv, sbi, cap-8*	*sak*	*icaD*	*sarA, efb*	1	Q-IIIA-t985	ST5
Wenzhou strain									
29	*hla, hlb, seg*	*fnbA, clfB, ebpS, cna, fnbB*	*hlg, sbi, cap-8, map*	*ssp, sak*	*icaD, icaA*	*sarA, efb*	1	J-I-t189	ST188
30	*hla*	*clfB, ebpS, cna*	*hlgv*, *sbi, cap-8*	*ssp*	*icaD*		2	A1-IIIA-t030	ST239
31, 32, 33	*hla, hlb, sea, seg,*	*fnbA, clfA, clfB, ebpS, cna*	*hlg, sbi, cap-8, map*	*ssp, sak*	*icaD, icaA*	*sarA, efb*	11	A1-IIIA-t030	
35, 40, 42, 43, 50, 51, 53	*hla, hlb, sea,*	*fnbA, clfA, clfB, ebpS, cna*	*hlg, sbi, cap-8, map*	*ssp, sak*	*icaD, icaA*	*sarA, efb*	1	A1-IIIA-t030	
52	*hla*	*clfB, ebpS, cna*	*hlg, cap-8*	*sak*	*icaD*	*sarA*	2	A1-IIIA-t030	
34	*hla, hlb*	*fnbA, clfA, clfB, bbp*	*hlg, cap-8, map*	*ssp, sak*	*icaD, icaA*	*sarA, efb*	1	H-IIIA-t2505	ST239
36, 37, 38, 39	*hla, hlb, sea,*	*fnbA, clfA, clfB, ebpS, cna*	*hlg, sbi, cap-8, map*	*ssp, sak*	*icaD, icaA*	*sarA, efb*	1	A2-IIIA-t030	ST239
41, 44, 46	*hla, hlb, sea,*	*fnbA, clfA, clfB, ebpS, cna*	*hlg, sbi, cap-8, map*	*ssp, sak*	*icaD, icaA*	*sarA, efb*	1	B-IIIA-t030	ST239
45	*hla*	*fnbA, clfB, ebpS, cna*	*hlg, sbi, cap-8*	*ssp, sak*	*icaD*	*efb*	2	B-IIIA-t030	
47	*hla, hlb, sea*	*clfB, cna*	*hlg, sbi, cap-8*	*sak*	*icaD, icaA*	*sarA*	1	A4-IIIA-t030	ST239
48	*hla, hlb, sea*	*fnbA, clfA, clfB, ebpS, cna*	*hlg, sbi, cap-8, map*	*ssp, sak*	*icaD, icaA*	*sarA, efb*	1	G-IIIA-t030	ST239
49	*hla, hlb, sea*	*fnbA, clfA, clfB, ebpS, cna*	*hlg, sbi, cap-8, map*	*ssp, sak*	*icaD, icaA*	*sarA, efb*	1	A3-IIIA-t030	ST239

* MLST (multilocus sequence typing) was performed on representative isolates for each PFGE-SCC*mec*-*spa* type;

? nt: nontypable; *agr*: accessory gene regulator; PFGE: pulsed-field gel electrophoresis; SCC*mec*:

staphylococcal chromosomal cassette *mec*; *spa*: staphylococcal protein A.

### agr Allele Distribution

Of the 53 isolates, 38 (16 from Shanghai and 22 from Whenzhou) were *agr-1*, 8 (5 Shanghai isolates and 3 Whenzhou isolates) were *agr-2*, and 7 (all from Shanghai) were *agr-3*. None was positive for *agr-4* (Table 4).

### MLST

A total of 27 isolates representative of each PFGE-SCC*mec*-*spa* profile were studied by MLST. And six ST types such as ST239, ST5, ST630, ST1, ST284 and ST188 were generated. ST239 was the most prevalent type (55.6%, 15/27), including A1 or A2 or A3 or A4 or B or G-IIIA-t030, A5 or D1-IIIA-t037, C-IIIB-t037, A1-IIIA-t2505, H-IIIA-t030, I or P1 or P2-III-t037, K-III-nt and L2-III-t002. The association between the other 5 ST types and PFGE-SCC*mec*-*spa* types were displayed in Table 4.

## Discussion

*S. aureus* produces numerous extracellular proteins which involve in the ability of this organism causing disease in the mammalian host. In this study, we detected six groups of pathogenic genes for 53 clinical MuH MRSA isolates from Shanghai and Wenzhou regions, China (Table 2). The first of these were the genes involved in toxin mediated disease. Previous study showed toxins encoded by *sea* and *sec* tend to generate higher immune responses resulting in host tissue damage than do other enterotoxins [16]. However, we did not find the existence of *sec* in Wenzhou isolates. It is generally believed that the existence of the enterotoxin gene cluster (*egc*, containing *seg* and *sei*) is not related with severe infections, but probably contributes to the colonization potential of an *S. aureus* strain [16], [17], [18]. Because the toxins transcripted by the *egc* element appear to be generated in lower amounts compared to the other well-studied enterotoxins which may permit strains carrying the *egc* determinants to live together with healthy hosts [17]. However, Morgan’s report showed the products of *egc* may play a part in some cases, especially in immuno-compromised patients [19]. Due to the genes *seg* and *sei* being located on *egc* element [20], the combined occurrence of the toxin gene pair can generally be observed. Notably, 25.0% (5/20) *seg-*positive isolates were not confirmed with the fixed *seg*-*sei* combination in our study. The *sed* and *sej* genes are encoded by a plasmid pIB485 [21]. However, the coexistence of the two determinants cannot also be certified with 2 *sej*-positive isolates (Table 4). The possible explanation for these opposite results is the existence of still-unknown variants of *sei* and *sed*.

The second group consisted of the determinants involved in attachment (*fnbA*, *fnbB*, *clfA*, *clfB*, *cna*, *bbp* and *ebpS*). The expression products of these genes were termed MSCRAMMs (microbial surface components recognizing adhesive matrix molecules). A great majority of our strains harbored the genes encoding FnbA, ClfA, ClfB, Cna and EbpS (Table 3), which may make them possess the ability to bind to fibronectin, fibrinogen and fibrin [22], collagen substrates and collagenous tissues [23], and soluble tropoelastin [24]. There is evidence that Bbp is a key factor in bone and joint infections produced by *S. aureus* [25], [26]. However, the positive rate of *bbp* implied most MuH MRSA strains of our collection might not have the ability to cause those diseases.

The third group included the genes *pvl*, *hlg*/*hlgv*, *cap-5*, *cap-8*, *map* and *sbi*. The toxins encoded by *pvl* and *hlg* are leukotoxic for neutrophils and macrophages [27]. Capsular polysaccharide (Cap) can protect the bacterium from phagocytic uptake and increases microbial virulence. Map may potentiate *S. aureus* survival by affecting protective cellular immunity [28], [29]. Sbi has the ability to hinder phagocytosis and is implicated in blood coagulation [30]. In this study, we did not see any significant difference in the prevalence of this group of genes except *pvl* between the Shanghai and Wenzhou strains (Table 3).

Ssp can degrade host cell receptors and/or bacterial adhesins, and promote the spread and transmission of infection [31]. Sak may mediate bacterial invasion into the host tissues and enhance bacterial resistance to phagocytosis [32]. According to Table 3, most of our isolates may have the functions mentioned above.

The genes *icaA* and *icaD* belong to *ica* operon (*icaADB* and *C*), which is revealed to induce polysaccharide intercellular adhesin (PIA, associated with biofilm formation) synthesis in staphylococcus. However, expression of *icaA* alone leads only to low production of PIA. It was demonstrated that coexpression of *icaA* with *icaD* will promote the biosynthesis of capsular polysaccharide [33]. In this study, a small part of our strains were negative for *icaA* (Table 3). This phenomenon may be the deficiency of *icaA* gene or the existence of point mutations in the primer binding sites causing a negative PCR reaction.

It has been hypothesized that Efb might benefit the bacterium by interacting with fibrinogen and preventing the clotting process, thereby delaying the healing process [34]. *sar* (containing 3 transcripts designated *sarA*, *sarB* and *sarC*) is a global regulatory locus, and controls the production of many virulence factors in *S. aureus*. Among which the *sarA* encodes the major effector molecule [35]. *ACME* can encode an arginine deiminase pathway and an oligopeptide permease system that could enhance the ability of *S. aureus*, especially for USA300 clone, to grow and survive within the host [36]. Table 3 showed the genes *efb* and *sarA* were prevalent in most our strains; however, no isolate studied possessed the founction of *ACME* gene.

*agr* is another important global regulatory locus controlling the production of most staphylococcal exoproteins. In *S*. *aureus*, four different *agr* alleles have been described and *agr* is regarded as a slowly evolving genetic marker to investigate hospital-acquired MRSA [37]. A report by Van Leeuwen et al. [38] suggested that the *agr-1* is the most prevalent *agr* group in MRSA isolates. We found similar in our isolates (71.7%, 38/53) (Table 4) and by Liu et al. [39] (96.4%, 134/139) in Beijing MRSA strains. Besides *agr-1*, 28.3% (15/53) of our strains belonged to *agr-2* or *agr-3*. Previous investigation has demonstrated the association between *agr-3* and TSST-1 [40], whereas the findings of this study revealed that only 6 of 14 *tst*-positive strains were *agr-3*, and the remainder belonged to *agr-1* (7 isolates) and *agr-2* (1 isolates) (Table 4). Likewise, the data reported by Ben Nejma et al. [41] could also not reveal the relationship between *agr-3* and this toxin.

Researches based on MLST exhibited that the predominant MRSA clone was ST239-MRSA in Asian countries besides Japan and South Korea (ST5). Yu et al. [42] and Yao et al. [43] reported that ST239-MRSA was the most commonly detected clone in MRSA obtained from Wenzhou, and Liu et al. [44] also discovered the prevalence of this clone in fourteen cities of mainland China, including Shanghai. Our study displayed the same results among the 27 MuH MRSA isolates representative (55.6%, 15/27). However, the ST5 isolates also accounted for 25.9% (7/27) of the representative strains (Table 4). Table 4 showed that the ST239-*agr1* (44.4%, 12/27), ST5-*agr1* (22.2%, 6/27) and ST239-*agr2* (18.5%, 5/27) were the common lineages in the two regions’ isolates.

For the determinants involved in toxin mediated disease, all the isolates from the two regions contained at least one gene of this group. However, there were 6 Shanghai strains and 4 Wenzhou isolates that did not carry any staphylococcal enterotoxin gene (Table 4). Table 4 shows there were 3 Shanghai isolates lacking genes involved in tissue invasion/penetration and the ‘others’ group genes (*sarA* and *efb*). In addition, 1 Shanghai isolate and 1 Wenzhou isolate were only absent from the genes divided into the ‘others’ group. In Shanghai and Wenzhou strains hosting ≤2 genes involved in toxin mediated disease, most of them also carried fewer other virulence genes (Table 4). In addition, Some MuH isolates yielded different virulence genes patterns even though they were of the same PFGE-SCC*mec*-*spa*-*agr*-ST type, and the same virulence determinants combination can belong to different genotype patterns (Table 4). In respect to these results, the possible explanation may be that some virulence determinants are located on mobile genetic elements and can be horizontally transmitted among bacteria.

In summary, our study showed there were some differences in virulence profiles between MuH MRSA isolates from Shanghai and Wenzhou, and the differences mainly existed in the genes *sea*, *sec*, *seg*, *sei*, *tst*, *cna* and *pvl*. The results of MLST and *agr* typing displayed that the two regions’ isolates were genetically less diverse.
